# Implementation of Digital Chest X-Ray with Computer-Aided Detection for Tuberculosis Screening Among Persons with Advanced HIV Disease in Maputo, Mozambique: A Retrospective Cohort Analysis

**DOI:** 10.3390/diagnostics16152393

**Published:** 2026-07-30

**Authors:** Maria Ruano Camps, Bendita Jose, Pereira Zindoga, Aleny Couto, Celia Cumbe, Gil Muvale, Rosa Bene, Admilson F. Cossa, Adrienne E. Shapiro, Jeff Lane, Florindo Mudender, Edy Nacarapa

**Affiliations:** 1 I-TECH Mozambique “International Training & Education Center for Health”, Bairro Sommerschield, Avenue Cahora Bassa N# 106, Maputo P.O. Box 1102, Mozambique; mariar@itech-mozambique.org (M.R.C.); celiac@itech-mozambique.org (C.C.); rbene@itech-mozambique.org (R.B.); admilsonc@itech-mozambique.org (A.F.C.); florindom@itech-mozambique.org (F.M.); 2 Centro de Referência de Alto-Maé “CRAM”, Maputo City Health Service, MoH, Maputo P.O. Box 264, Mozambique; gilmuvale@gmail.com; 3 National TB Program, Ministry of Health, Maputo P.O. Box 264, Mozambique; beneditajose1@gmail.com (B.J.); pzindoga@leap-global.org (P.Z.); 4 National Directorate of Public Health (DNSP), Ministry of Health, Maputo P.O. Box 264, Mozambique; coutoaleny@gmail.com; 5 Department of Global Health, University of Washington, 3980 15th Ave NE, Seattle, WA 98195, USA; aeshapir@uw.edu (A.E.S.); lanej3@uw.edu (J.L.)

**Keywords:** tuberculosis, HIV, advanced HIV disease, artificial intelligence, digital chest X-ray, computer-aided detection, implementation science, Mozambique, retrospective cohort, programmatic data

## Abstract

**Background:** Tuberculosis (TB) remains the leading cause of death among persons with advanced HIV disease (AHD) in high HIV-burden settings. Digital chest X-ray (dCXR) with computer-aided detection (CAD) is a promising tool to overcome human resource constraints and improve TB case detection. This study evaluates the real-world implementation and performance of dCXR/CAD for TB screening within a specialized AHD clinic in Maputo, Mozambique. **Methods:** We conducted a retrospective cohort analysis of 487 new AHD patients at Centro de Referência do Alto Maé (CRAM) from October 2023 to September 2024. Of these, 238 underwent dCXR with CAD interpretation. All patients underwent systematic TB screening according to Ministry of Health (MoH) guidelines. Using the recorded diagnosis of TB (bacteriologically confirmed or clinically diagnosed) as the reference standard, we calculated the sensitivity, specificity, positive predictive value (PPV), and negative predictive value (NPV) of the nationally adopted CAD threshold (≥0.5). **Results:** Among 238 AHD patients screened with CAD, 116 (49%) were diagnosed with TB. At the ≥0.5 threshold, sensitivity was 50% (58/116; 95% CI: 41–59), specificity 92% (112/122; 95% CI: 85–96), PPV 85% (58/68; 95% CI: 75–92), and NPV 65.9% (112/170; 95% CI: 58–73). TB diagnosis rates increased sharply with CAD score: 30% (43/143) in normal, 52% (15/27) in abnormal non-suggestive, and 85% (58/68) in suggestive cases. Bacteriological confirmation was low across all groups (19–26%), reflecting reliance on clinical diagnosis. **Conclusions:** Integrating dCXR/CAD into AHD care is feasible and identifies a high TB burden. However, at the adopted threshold of ≥0.5, CAD demonstrated high specificity but low sensitivity (50%) in this population, missing half of all TB cases. These findings suggest that CAD functions better as a confirmatory decision-support tool than a standalone screening test in AHD. Threshold optimization for this specific population warrants prospective evaluation. Implementation challenges including fragmented systems and lack of dedicated human resources must be addressed to realize CAD’s full potential in TB programs.

## 1. Introduction

### 1.1. The Dual Epidemic of TB and HIV in Mozambique

Mozambique faces a severe dual epidemic of HIV and tuberculosis (TB). With an estimated population of 33 million, the country has an adult HIV prevalence of 12.5% and an estimated TB incidence of 361 per 100,000 population [1]. HIV dramatically increases the risk of developing active TB, accelerates its progression, and complicates its diagnosis and treatment. Among people living with HIV (PLHIV), those with advanced HIV disease (AHD) defined by the World Health Organization (WHO) as a CD4 cell count < 200 cells/mm^3^ or the presence of a WHO stage 3 or 4 defining illness are at the highest risk of mortality, with TB being the leading cause of death [2,3].

Despite scale-up of antiretroviral therapy (ART), late presentation to care for HIV and recurrent disengagement from treatment continue to pose significant challenges. In Mozambique, early mortality after ART initiation is high, often driven by undiagnosed opportunistic infections (OIs), principally TB [4]. The national TB case notification rate (334/100,000) closely mirrors the estimated incidence, suggesting a strong detection effort, yet gaps persist, particularly among high-risk subgroups like patients with AHD [1]. The low proportion of bacteriologically confirmed TB (43%) indicates substantial reliance on clinical diagnosis, which can be non-specific in AHD, leading to both over- and under-treatment [1].

### 1.2. Challenges in TB Diagnosis Among Persons with AHD

Diagnosing TB in AHD is notoriously difficult. Classic symptoms may be absent, and the radiological presentation on chest X-ray (CXR) can be atypical, non-cavitary, and disseminated, often resembling other pulmonary conditions common in HIV. Often, the chest X-ray may appear entirely normal [3,5]. Sputum-based diagnostic tests, such as Xpert MTB/RIF, have lower sensitivity in this population, especially in those with low CD4 counts and extrapulmonary or paucibacillary disease [6,7]. This diagnostic complexity necessitates a high index of suspicion and a comprehensive screening approach that integrates clinical, radiological, and microbiological tools.

### 1.3. The Role of Digital Chest X-Ray and Computer-Aided Detection

Digital chest X-ray (dCXR) has emerged as a critical screening tool for TB. It is fast, non-invasive, and can detect abnormalities suggestive of pulmonary TB even in subclinical cases. When paired with artificial intelligence (AI) in the form of computer-aided detection (CAD) software qXR v4.0 (Qure.ai, India), dCXR can automate image interpretation, providing a standardized, reproducible “score” or probability of TB. This is particularly valuable in settings with a shortage of skilled radiologists, by helping to scale and decentralize screening and prioritize individuals for further diagnostic evaluation [8].

The ideal CAD score threshold may vary by setting, depending on clinical population characteristics, the background burden of non-TB lung disease, and the intended role of CAD in the diagnostic pathway. The WHO now recommends CAD as an alternative to human reading for screening and triage [9].

However, its performance and optimal implementation in real-world, high-HIV-burden clinical settings outside of controlled study conditions require further evaluation. Key questions remain about the optimal CAD score threshold for a given setting, its performance in AHD populations where TB presentation and radiological findings are frequently atypical—or entirely absent—and the practical challenges of integrating this technology into routine clinical workflows.

### 1.4. The CRAM Clinic as a Natural Implementation Setting

The Centro de Referência do Alto Maé (CRAM), managed by the International Training and Education Center for Health (I-TECH) in Maputo, serves as a centre of excellence for the management of AHD in Mozambique. It provides rapid, comprehensive, same-day screening and initiation of treatment for major OIs, including Cryptococcal meningitis [10] and TB [11,12]. The clinic’s patient population consists of individuals referred for very low CD4 counts, ART treatment failure requiring HIV-1 genotyping [13], or severe comorbidities, and represents a group at extreme risk for TB and mortality [14,15,16]. In 2023, with USAID support, CRAM installed a dCXR machine and CAD software, making it the only primary-level outpatient facility in Maputo city with this capacity.

### 1.5. Study Rationale and Objectives

Recent studies have evaluated CAD among PLHIV in various settings. Burke et al. demonstrated that CAD combined with urine LAM improved inpatient TB diagnosis [17]. MacPherson et al. found CAD screening increased TB case detection among adults with cough in Malawi [18]. Olotu et al. reported high CAD uptake in Mozambican prisons [19]. Moodley et al. and Kagujje et al. evaluated CAD performance in primary care and among persons with prior TB, respectively [20,21]. Qin et al. and Nzimande et al. provided multi-country and regional validation data [22,23].

However, none of these studies focused specifically on persons with advanced HIV disease (CD4 < 200 cells/mm^3^ or WHO stage 3/4), a group with atypical radiological presentations and particularly high TB burden. Moreover, while efficacy studies have demonstrated CAD’s accuracy in controlled settings, data on its real-world effectiveness within routine, resource-constrained TB/HIV programs remain scarce. Our study addresses both gaps by providing the first dedicated evaluation of CAD performance and threshold selection in an AHD cohort in a high-burden African setting, using routinely collected programmatic data. Guided by implementation science frameworks that emphasize understanding context, processes, and outcomes [24], this retrospective evaluation:

1. Described the integration process of dCXR/CAD into the routine clinical workflow of a specialized AHD clinic.
2. Evaluated the diagnostic yield of dCXR/CAD among patients with AHD, stratified by CAD result category.
3. Assessed the performance of the nationally adopted CAD threshold (≥0.5) in the AHD population.
4. Listed operational challenges, facilitators, and insights from the first year of implementation.

Rather than a formal diagnostic accuracy study, this analysis provides critical programmatic evidence to inform national policy on CAD use, optimize clinical algorithms for TB screening in AHD, and improve implementation strategies for novel diagnostic technologies in low-resource settings.

## 2. Materials and Methods

### 2.1. Evaluation Design and Setting

This was a retrospective cohort analysis of routinely collected clinical and programmatic data. The study was conducted at the CRAM clinic, a specialized outpatient service for AHD within the Alto Maé Health Center in Maputo City, Mozambique. CRAM operates as a referral center for complex HIV cases from across the city’s primary health facilities and hospitals [11].

### 2.2. Evaluation Population and Cohort

The analysis focused on one cohort screened for TB over a 12-month period (October 2023–September 2024), consisting of all new HIV+ patients admitted to CRAM during the study period (*N* = 487). Admission criteria included CD4 count < 100 cells/mm^3^ (or <200 if symptomatic), Kaposi’s sarcoma (KS), second-line ART failure, or complex comorbidities. To contextualize CAD performance, we used data from 2695 patients undergoing dCXR at Alto Maé Health Centre, a primary-level facility serving both HIV-positive and HIV-negative patients, as a proxy for the general population undergoing CXR in this setting. This reference cohort includes the AHD patients described in this study, as both groups used the same machine during the same period.

### 2.3. The Intervention: dCXR with CAD Integration

The intervention was the integration of systematic dCXR screening with CAD interpretation into the clinic’s existing AHD care package.

A standalone digital X-ray machine was connected to qXR v4.0 (Qure.ai, India), one of six CAD software systems approved by the WHO for TB screening [25]. The CAD software analyses posterior-anterior dCXR images and assigns a score from 0 to 1, indicating the probability of TB-associated abnormalities. According to WHO TDR guidance on CAD calibration studies [25], this represents a triage threshold intended to prioritize individuals for confirmatory testing. The ≥0.5 threshold is the manufacturer’s default setting (qXR v4.0). The National TB Program (PNCT) adopted this threshold pending local calibration. We evaluated the performance of this threshold against recorded diagnosis of TB.

For the AHD cohort, dCXR was part of the routine same-day admission assessment. However, CAD results were not integrated into the real-time clinical workflow and did not formally alter the diagnostic pathway. Clinical decisions, including the use of TB-LAM, Xpert MTB/RIF, and treatment initiation, were made by the clinical team based on national protocols, clinical evaluation, laboratory results, and direct review of the chest X-ray image, independent of the CAD score.

### 2.4. Data Sources and Variables

Data were extracted from the clinic’s electronic medical records and paper-based registers, which are part of routine national HIV and TB monitoring systems.

*Independent variables*: CAD result category (Invalid, Normal, Abnormal not suggestive of TB [score < 0.5], Abnormal suggestive of TB [score ≥ 0.5]).

*Primary outcome*: Recorded diagnosis of TB (bacteriologically confirmed or clinically diagnosed).

*Secondary outcome*: Bacteriological confirmation of TB (by Xpert MTB/RIF or culture).

### 2.5. Data Analysis

The performance analysis of dCXR/CAD was conducted retrospectively, using recorded TB diagnoses as the reference standard. Descriptive statistics were used to characterize the cohort. Analysis was primarily focused on diagnostic yield and CAD performance metrics. For each CAD result category, we calculated: (1) Proportion of patients with recorded diagnosis of TB; and (2) Proportion of those with bacteriological confirmation. We also calculated sensitivity, specificity, PPV, and NPV for the CAD ≥ 0.5 threshold using the recorded diagnosis of TB as the reference standard.

Traditional sensitivity/specificity calculations against a perfect gold standard were not feasible, because most patients had a clinical diagnosis of TB and did not undergo exhaustive confirmatory testing (e.g., culture). Instead, the proportion of patients ultimately diagnosed and treated for TB was used as a pragmatic measure of CAD’s programmatic utility.

Data were managed and analysed using Microsoft Excel and Stata version 18.0.

### 2.6. Evaluation Metrics

This evaluation is not a diagnostic accuracy study of the dCXR/CAD technology. Rather, it is a retrospective evaluation of an implementation of this technology to understand how CAD scores, obtained in an uncontrolled, operational setting, reflect the TB diagnoses made by clinicians in this context. The metrics presented are reference-dependent and should be interpreted as programmatic performance indicators, not as conventional diagnostic accuracy measures.

Diagnostic performance was assessed using four standard metrics and a reference standard defined as the recorded diagnosis of TB, whether bacteriologically confirmed or clinically diagnosed. Sensitivity, calculated as TP divided by the sum of TP and FN, measures the proportion of TB cases correctly identified by CAD. Specificity, calculated as TN divided by the sum of TN and FP, measures the proportion of non-TB cases correctly identified as negative. Positive Predictive Value (PPV), calculated as TP divided by the sum of TP and FP, reflects the proportion of CAD-positive results that were true TB cases, and Negative Predictive Value (NPV), calculated as TN divided by the sum of TN and FN, reflects the proportion of CAD-negative results that were true non-TB cases. In all formulas, TP refers to true positives, TN to true negatives, FP to false positives, and FN to false negatives.

### 2.7. Ethical Considerations

Ethical clearance was obtained from the Mozambican National Bioethics Committee for Health (IRB0002657—Comité Nacional de Bioética para a Saúde, nr: 21/CNBS/2025) and the permission to perform this evaluation was also obtained from the Health Service of Maputo city (Serviço de Saúde da Cidade, N/Ref. no. 7002/2103/SSCM/2024).

## 3. Results

### 3.1. Context and Cohort Description

**Figure 1:**
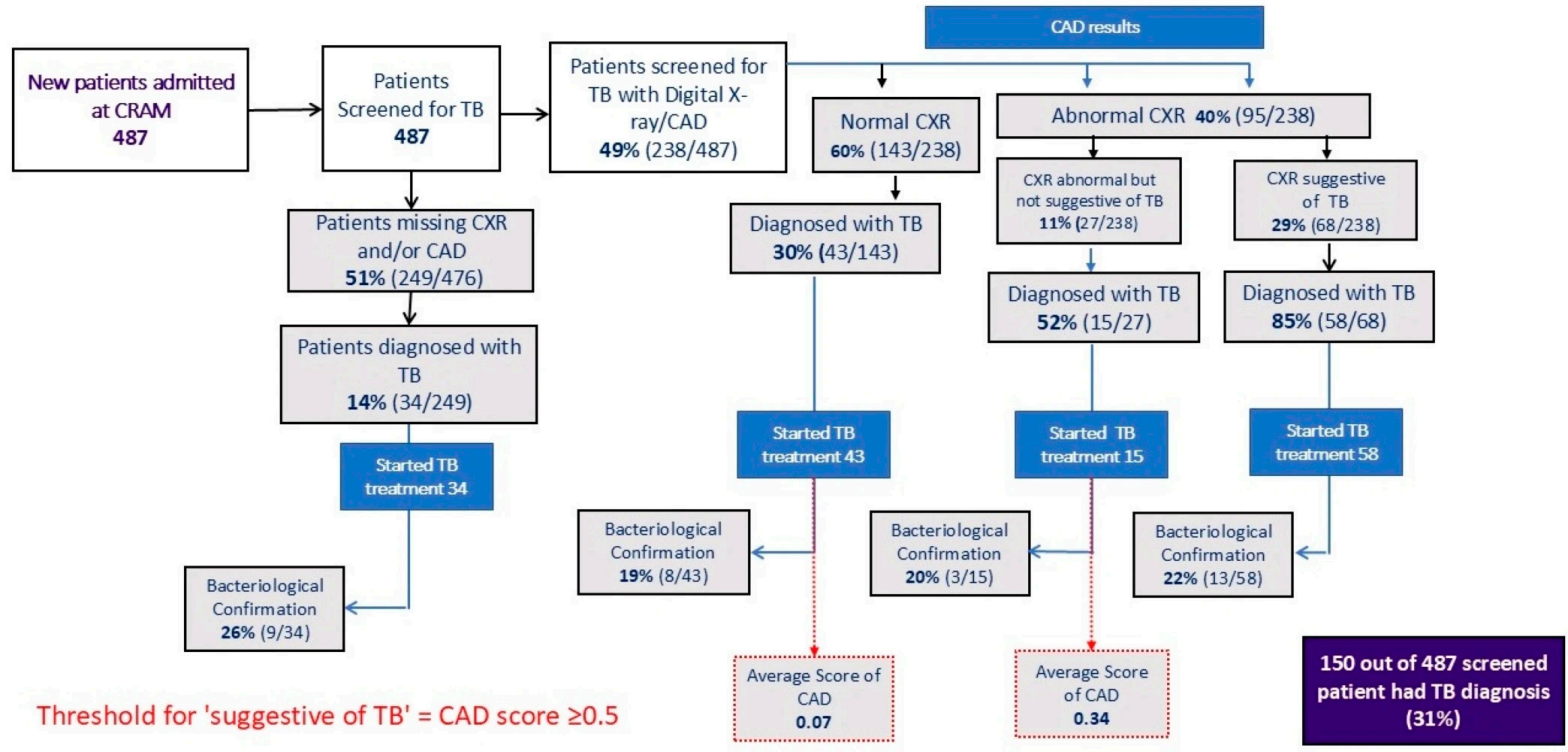
All TB cases—New patients admitted at CRAM (12 months). **Overall Burden:** 31% (150/487) of all new AHD patients were diagnosed with TB.

Between October 2023 and September 2024, 487 new patients were admitted to CRAM. The cohort comprised 53% men, with a median CD4 count at admission of 211 cells/mm^3^. The main reasons for admission were low CD4 count (40%), cryptococcal disease (11%), Kaposi’s sarcoma (23%), treatment failure (10%), and other medical complications (16%). The median CD4 of 211 cells/mm^3^ is higher than typically expected in an AHD cohort, which is partly explained by the fact that 23% of patients were admitted for Kaposi’s sarcoma, condition that defines AHD by WHO clinical stage regardless of CD4 count. Notably, 150 out of 487 (32%) of all new enrolees were ultimately diagnosed with TB and started on treatment, underscoring the high TB burden in this population (Figure 1).

### 3.2. dCXR/CAD Screening Uptake

Among 487 new AHD patients, 238 (49%) underwent dCXR with CAD reading. The remaining 51% either did not receive a dCXR or their dCXR was not processed by the CAD software due to operational constraints (Figure 1).

### 3.3. dCXR/CAD Performance and TB Detection

#### 3.3.1. CXR/CAD Positivity in the Local General Population—Contextual Reference

In the general population (both PLHIV and HIV-negative patients) screened at the nearby primary health facility (*N* = 2695), 90% (2425/2695) had valid dCXR/CAD results. Of those with a valid result, 43% (1035/2425) were interpreted as abnormal. Among these abnormal results, 54% (564/1035) were flagged as suggestive of TB (CAD score ≥ 0.5), representing 23% (564/2425) of all valid dCXR/CAD. This cohort provides local contextual data on CAD positivity rates among the general population accessing radiography services in Maputo City and should not be interpreted as a formal standard. As both groups used the same machine during the same period, the AHD patients described in this study are included within this reference cohort; the comparison therefore serves only as a local reference for CAD positivity rates in the Maputo context, rather than a formal comparison between the two cohorts.

#### 3.3.2. TB Detection by CAD Result Category in AHD Patients at CRAM

Among the 238 AHD patients screened with CAD at CRAM: 60% (143/238) had a normal CXR (score typically < 0.1); 11% (27/238) had an abnormal CXR not suggestive of TB (score < 0.5); 29% (68/238) had a CXR suggestive of TB (score ≥ 0.5).

TB diagnosis rates increased with CAD score (Table 1): 30% (43/143) among those with a normal result, 52% (15/27) among those with an abnormal non-suggestive result, and 85% (58/68) among those with a suggestive result. A chi-square test for trend confirmed a statistically significant positive association between higher CAD score categories and TB diagnosis (*p* < 0.001).

Bacteriological confirmation was similarly low across all groups: 19% (8/43) in the normal group, 20% (3/15) in the non-suggestive group, and 22% (13/58) in the suggestive group. This confirms the high rate of clinical diagnosis of TB in AHD population (Table 1).

### 3.4. Diagnostic Performance of CAD ≥ 0.5 Threshold

The diagnostic performance of the manufacturer-recommended CAD threshold (≥0.5) was evaluated against the recorded diagnosis of TB. Table 2 presents the 2 × 2 contingency table with case and control classifications.

At the current threshold of ≥0.5, CAD missed 58 of 116 TB cases (false negatives), resulting in a sensitivity of only 50%. However, among the 68 patients flagged as CAD-positive, 58 (85%) were true positives, highlighting a relatively high PPV (85%). The relatively low NPV (66%) indicates that a negative CAD result does not reliably rule out TB in this AHD population. The high specificity (92%) means that false positives are relatively less common (8%).

The mean CAD score across all patients varied by result category: 0.07 among those with a normal result (*n* = 143), 0.34 among those with an abnormal but non-suggestive result (*n* = 27), and 0.83 among those with a result suggestive of TB (*n* = 68).

## 4. Discussion

This retrospective implementation analysis of the first year of dCXR/CAD deployment at a reference AHD clinic in Mozambique provides real-world evidence on the promise and pitfalls of this technology in a high-burden, resource-constrained setting.

### 4.1. Integration of dCXR/CAD into Routine AHD Care

The reach of CAD screening among AHD patients was 49%, indicating that while the tool was adopted, its consistent application was limited. For AHD patients, the integration was into a complex, multi-faceted same-day assessment for high risk and sometimes critically ill individuals. Operational barriers such as the non-integrated system and lack of dedicated staff directly limited reach. This underscores a key implementation principle: even highly effective tools fail if the workflow integration is suboptimal [17]. Successful adoption requires not just technology but also process redesign and dedicated human resources to manage it.

### 4.2. CAD Performance in Context: Confirmatory Rather than Triage Tool

The performance of CAD in this cohort reflects a pattern more consistent with a confirmatory adjunct than a triage tool. In a population where advanced HIV disease and a high background TB burden converge, the pre-test probability of TB is naturally elevated. A positive CAD result with a PPV of 85% adds meaningful diagnostic weight, when clinical evaluation already points towards TB, supporting the decision to initiate treatment. However, the low sensitivity of 50% means that a negative result carries limited reassurance. Among AHD patients, 30% of those with a normal CAD result and 52% of those with an abnormal but non-suggestive result were ultimately diagnosed with TB. This reflects a well-recognized phenomenon: TB presentation in severely immunocompromised patients is frequently atypical, subtle, disseminated, or mimicking other opportunistic infections, and CAD algorithms trained on general population data may lack sensitivity for these patterns [5,10]. A non-suggestive CAD result should therefore not reduce clinical suspicion in a patient with advanced HIV disease.

### 4.3. Clinical Diagnosis Requires a Multi-Test Approach

Importantly, diagnosis in this population must rest on a constellation of findings rather than any single result. The low bacteriological confirmation rates in this cohort (19–22%) reinforce that clinicians need to integrate clinical signs, symptoms, and complementary tests, including urine TB-LAM alongside CAD output [26].

### 4.4. Threshold Recalibration and Risk-Stratified Implementation in AHD

The high TB diagnosis rate (52%) among patients with an abnormal but non-suggestive result (mean CAD score 0.34) suggests a potential rationale for exploring a lower threshold in this population. For instance, lowering the threshold from 0.5 to 0.4 would, by definition, be expected to increase sensitivity at the cost of specificity, a trade-off that might be acceptable if the goal is to maximize case finding. However, as this was not evaluated in our study, this remains a hypothetical example, and formal threshold optimization for this specific population warrants prospective evaluation. Context-specific calibration of AI thresholds remains a recognised challenge in global health implementation [27].

The findings highlight the tension between fidelity to the manufacturer-recommended threshold (≥0.5) and the need for local adaptation [28]. For AHD patients, a lower CAD threshold combined with a predefined intensive workup protocol Xpert, urine TB-LAM, ultrasound could formalize this adaptation [29].

### 4.5. Challenges and Sustainability

The identified challenges point to several threats to long-term sustainability. First, system fragmentation represents a critical bottleneck, as the disconnect between the X-ray machine and the CAD software necessitates manual image transfers and creates dependence on stable connectivity; therefore, investment in integrated, plug-and-play systems is important for scaling [30]. Second, a human resource gap exists because technology does not replace human input but rather reallocates it. A designated staff role for dCXR and CAD management is critical for consistent use and data quality [9]. Third, the lack of quality assurance systems poses a significant risk. For CAD to be trusted and improved, a national monitoring system and periodic recalibration against local patient outcomes are needed [22,25].

### 4.6. Limitations

This study has limitations inherent to retrospective programmatic data, including missing data, particularly for patients not receiving CAD. The absence of a culture-confirmed gold standard means the sensitivity and specificity estimates we report are reference-dependent and may overestimate performance if clinical diagnoses include false positives or underestimate it if subclinical cases were missed. Although CAD results were not integrated into real-time clinical decisions, the potential for incorporation bias cannot be entirely excluded. Patients who underwent dCXR/CAD had a substantially higher TB diagnosis rate than those who did not (49% vs. 14%). Although dCXR was intended as a routine first-visit assessment for all patients, operational barriers such as power outages, CAD synchronization failures, or the temporary absence of a trained technician prevented it in some cases. In these situations, patients with higher clinical suspicion of pulmonary TB may have been more likely to have CXR ensured at a subsequent visit, while those with lower suspicion may not have had it performed at all. If this clinical prioritization occurred, it would have contributed to the observed selection bias and likely inflates PPV estimates. Detailed data on individual diagnostic tests performed (Xpert MTB/RIF, urine TB-LAM, culture, and extrapulmonary evaluation) are not available in a structured format, limiting full characterization of the diagnostic pathway. A stratified analysis by CD4 count was not performed, as this study aimed to describe CAD performance in the AHD population as a whole; this represents a worthwhile avenue for future research. Finally, our findings apply to adults with AHD in a single urban site; generalizability to children with HIV or to more remote settings requires separate evaluation, though CRAM is likely representative of urban AHD cohorts in high-burden African settings [22,25].

The reference standard was based on clinical diagnosis, which was informed by direct review of CXR. This creates the potential for incorporation or review bias, and therefore, our estimates of sensitivity and specificity should not be interpreted as definitive test accuracy against an independent gold standard.

No formal, granular image quality-control assessment (e.g., distinguishing between motion artifacts, incorrect positioning, or other technical issues) was conducted as part of this retrospective evaluation. The CAD software automatically flags images as “invalid” based on its internal algorithms, but a specific breakdown of the reasons for rejection is not available in the programmatic dataset. The lack of a formal human- or system-based QC process is a limitation of our implementation analysis.

## 5. Conclusions and Recommendations

The implementation of dCXR/CAD at CRAM confirms the high yield of systematic TB screening in AHD populations, while also revealing how its current application, using a general-population threshold, risks missing a significant number of TB cases among the most vulnerable patients while generating false positives in others. Based on these results and our experience implementing this technology, we recommend:

- For national programs: Conduct an urgent review of the CAD score threshold for PLHIV, particularly those with AHD. Consider establishing a lower, more sensitive threshold for this group, which should be validated through prospective studies.
- For clinic managers: Integrate dCXR/CAD findings into a composite clinical algorithm for AHD. A non-suggestive CAD result should not end the screening process for AHD patients. Advocate for and allocate a dedicated staff position to manage the CAD workflow.
- For implementers and donors: Prioritize funding for integrated dCXR/CAD systems over fragmented components. Support the development of national QA systems and operational research to continually evaluate and adapt CAD use in different patient populations.
- For future research: Prospective studies are needed to validate an optimized, HIV-specific CAD threshold and to evaluate the cost-effectiveness and impact on mortality of dCXR/CAD-based screening in AHD care packages.

In conclusion, dCXR/CAD shows real promise as a decision-support tool in AHD care. At the current threshold, a positive result can add meaningful diagnostic weight when TB is already clinically suspected, supporting timely treatment decisions. However, a negative or non-suggestive result should not reduce clinical suspicion in this population. Realising its potential will require threshold optimization, careful clinical integration, and a commitment to evaluate global tools against local, high-risk realities.

## Figures and Tables

**Table 1:** TB diagnosis and bacteriological confirmation by CAD result category in AHD patients.

CAD Result Category	Patients (*N*)	Mean CAD Score	Recorded Diagnosis of TB (*n*, %)	*p*-Value Chi-Square	Bacteriologically Confirmed (*n*, %)
No CXR/CAD Done	249		34/249 (14%)		9/34 (26%)
Normal	143	0.07	43/143 (30%)	*p* < 0.001	8/43 (19%)
Abnormal, Not Suggestive (score < 0.5)	27	0.34	15/27 (52%)		3/15 (20%)
Abnormal, Suggestive of TB (score ≥ 0.5)	68	0.83	58/68 (85%)		13/58 (22%)
Total (with CAD)	238		116/238 (49%)		24/116 (21%)
COHORT TOTAL	487		150/487 (31%)		33/150 (22%)

Notably, bacteriological confirmation rates were similarly low (19–22%) across all CAD score categories.

**Table 2:** 2 × 2 contingency table—CAD ≥ 0.5 threshold vs. TB diagnosis (reference standard).

	TB Case (Recorded Diagnosis of TB)	Control (Not Diagnosed with TB)	Subtotal
CAD Suggestive (≥0.5)	58 (True Positive)	10 (False Positive)	68
CAD Not Suggestive (<0.5)	58 (False Negative)	112 (True Negative)	170
Subtotal	116	122	238 (Total)

Threshold “suggestive of TB” = CAD score ≥ 0.5. Diagnostic performance: Sensitivity 50% (95% CI: 41–59) | Specificity 92% (95% CI: 85–96) | PPV 85% (95% CI: 75–92) | NPV 66% (95% CI: 58–73).

## Data Availability

The data presented in this study are available on request from the corresponding author due to privacy constraints.

## Abbreviations

The following abbreviations are used in this manuscript:

1. Medical and Clinical Terms	
AHD	Advanced HIV Disease
ART	Antiretroviral Therapy
ARV	Antiretroviral
Bact	Bacteriologically
CAD	Computer-Aided Detection
CD4	Cluster of Differentiation 4 (type of immune cell)
CrAg	Cryptococcal Antigen
CXR	Chest X-Ray
dCXR	Digital Chest X-Ray
Dx	Diagnosis
EP TB	Extrapulmonary Tuberculosis
FASH	Focused Assessment with Sonography for HIV/TB
HF	Health Facility
KS	Kaposi’s Sarcoma
LAM	Lipoarabinomannan (TB antigen test)
LTFU	Lost to Follow-Up
MDR/RR-TB	Multidrug-Resistant/Rifampicin-Resistant Tuberculosis
OI	Opportunistic Infection
PLHIV	People Living with HIV
TB	Tuberculosis
TF	Treatment Failure
Tx	Treatment
VL	Viral Load
Xpert	GeneXpert MTB/RIF (molecular diagnostic test)
2. Organizations and Programs	
CDC	Centers for Disease Control and Prevention
C&T	Care and Treatment
CRAM	Centro de Referência do Alto Maé
HRSA	Health Resources and Services Administration
I-TECH	International Training and Education Center for Health
USAID	United States Agency for International Development
WHO	World Health Organization
3. Research and Data Terms	
Cohort	A defined group studied in research
EQA	External Quality Assessment
n/N	Number of cases/Total number in group
